# Use of posterior predictive checks as an inferential tool for investigating individual heterogeneity in animal population vital rates

**DOI:** 10.1002/ece3.993

**Published:** 2014-03-20

**Authors:** Thierry Chambert, Jay J Rotella, Megan D Higgs

**Affiliations:** 1 Department of Ecology, Montana State UniversityBozeman, Montana; 2 Department of Mathematical Sciences, Montana State UniversityBozeman, Montana

**Keywords:** Bayesian inference, dynamic heterogeneity, fixed heterogeneity, individual variation, *Leptonychotes weddellii*, model checking, posterior predictive checking, state-space models

## Abstract

The investigation of individual heterogeneity in vital rates has recently received growing attention among population ecologists. Individual heterogeneity in wild animal populations has been accounted for and quantified by including individually varying effects in models for mark–recapture data, but the real need for underlying individual effects to account for observed levels of individual variation has recently been questioned by the work of Tuljapurkar et al. (Ecology Letters, 12, 93, 2009) on dynamic heterogeneity. Model-selection approaches based on information criteria or Bayes factors have been used to address this question. Here, we suggest that, in addition to model-selection, model-checking methods can provide additional important insights to tackle this issue, as they allow one to evaluate a model's misfit in terms of ecologically meaningful measures. Specifically, we propose the use of posterior predictive checks to explicitly assess discrepancies between a model and the data, and we explain how to incorporate model checking into the inferential process used to assess the practical implications of ignoring individual heterogeneity. Posterior predictive checking is a straightforward and flexible approach for performing model checks in a Bayesian framework that is based on comparisons of observed data to model-generated replications of the data, where parameter uncertainty is incorporated through use of the posterior distribution. If discrepancy measures are chosen carefully and are relevant to the scientific context, posterior predictive checks can provide important information allowing for more efficient model refinement. We illustrate this approach using analyses of vital rates with long-term mark–recapture data for Weddell seals and emphasize its utility for identifying shortfalls or successes of a model at representing a biological process or pattern of interest.

We show how posterior predictive checks can be used to strengthen inferences in ecological studies. We demonstrate the application of this method on analyses dealing with the question of individual reproductive heterogeneity in a population of Antarctic pinnipeds.

## Introduction

In the last decade, ecologists have devoted growing effort to the investigation of individual heterogeneity in wild animal population vital rates (e.g., Cam et al. 2002, 2012; Steiner et al. 2010; Orzack et al. 2011). Theoretically, individual heterogeneity can be directly captured through individual covariates, but when such covariates are not available from field data or cannot easily be identified, latent individual differences can still be modeled as finite mixtures (Pledger et al. 2010) or as individual random effects (Royle 2008). Mark–recapture analyses including random effects have grown more popular in recent years thanks to methodological developments that allow straightforward model implementation in both Bayesian (Gimenez et al. 2007, 2009; King et al. 2009; Link and Barker 2010; Kéry and Schaub 2012) and likelihood frameworks (Gimenez and Choquet 2010). Although individual random effects can be incorporated in mark–recapture models solely to deal with violations of homogeneity and independence assumptions (Marzolin et al. 2011), they are often of direct biological interest (Cam et al. 2002; Cooch et al. 2002). In these models, the magnitude of underlying individual heterogeneity is estimated through the variance parameter of the distribution of individual effects.

Translating statistical inference about variance parameters into biologically meaningful conclusions is often difficult, mainly because it is challenging to attach practical meaning to the magnitude of a variance parameter, particularly on a transformed scale such as the logit. Traditional statistical tests with a null value of zero do not take into account whether the magnitude is practically meaningful even if it is statistically different from zero, or whether plausible values for the variance are in fact practically meaningful even if very small values cannot be ruled out given the data. This issue is particularly crucial to consider when evaluating evidence for whether underlying individual heterogeneity is important to include for explaining observed differences in individual lifetime performances (Cam et al. 2012). Indeed, previous work (Tuljapurkar et al. 2009) has shown that some degree of realized heterogeneity in individual performances, such as lifetime and reproductive success, is always expected, just by chance, even in the total absence of underlying individual heterogeneity in vital rates. This phenomenon has been referred to as dynamic heterogeneity and is the result of the stochastic nature of individual life trajectories that result from a sequence of binomial events (lives/dies; breeds/does not breed). Therefore, in addition to estimating the variance parameter representing the assumed heterogeneity, it is also important to address the question of whether underlying individual effects are actually needed to explain realized levels of individual variation. To address this issue, previous authors have used model-selection methods (e.g., Cam et al. 2012) based on information criteria such as the deviance information criterion (DIC; Spiegelhalter et al. 2002) and using Bayes factors (Link and Barker 2006) to compare the support for models with and without individual random effects. However, given that no wild population can be considered as entirely homogeneous, one can easily argue that the question of individual heterogeneity comes down to assessing the magnitude of variance rather than simply choosing between two models (homogeneity vs. heterogeneity). However, both the homogeneity and heterogeneity models can be useful to address a wide range of ecological questions, and we think that a more thorough investigation of the question of individual heterogeneity involves assessing (i) the implications of ignoring individual heterogeneity in terms of ecologically meaningful measures, and (ii) how the estimated magnitude of individual heterogeneity actually manifests in those ecologically meaningful measures. Posterior predictive checks provide a straightforward method for such thorough investigation and can be useful in the process of statistical inference by providing detailed information on how a model succeeds or fails at capturing some measures of biological interest (Gelman et al. 1996). We therefore propose posterior predictive model checks (Gelman et al. 2004) as an additional and important tool for further assessing the implications of excluding or including individual random effects in demographic models.

Posterior predictive checks are based on the comparison of the distribution of model-specific data replications with observed data. The approach is aimed at identifying and quantifying systematic discrepancies between a model and the observed data, while accounting for parameter uncertainty. The idea of model checking is not new in the population ecology literature (Lebreton et al. 1992; Williams et al. 2002), but classic goodness-of-fit tests developed for mark–recapture models are only useful to assess potential violations of basic independence assumptions and are limited to a few specific model classes (Cormack–Jolly–Seber and Jolly movement models; Williams et al. 2002). Posterior predictive checks allow an assessment of the performance of any model of interest at predicting any specific aspect of the data. Furthermore, in mark–recapture studies, model checking has traditionally not been used as an informative tool to help understand the implications of different models. Here, in the spirit of Gelman et al. (2004), we argue for incorporating model checking into the inferential process, as a way to identify the shortfalls and successes of specific models and thereby improve our understanding of biological processes of interest. In particular, by investigating the lack of fit of models that exclude individual heterogeneity, posterior predictive checks allow a direct and more relevant assessment of the issues of underlying vs. dynamic heterogeneity arguments. Keys to effective model checks are the use of graphical displays and the careful choice of discrepancy measures that are relevant to the scientific question of interest. For models of individual heterogeneity, we suggest deriving data quantities that directly represent the level of realized variation in individual performance and using histograms to compare the posterior predictive distribution of these quantities of interest to the values calculated from the observed data.

In the following sections, we describe the principle of posterior predictive checks and illustrate their use with a case study that investigates the magnitude of individual reproductive heterogeneity in a population of Weddell seals (*Leptonychotes weddellii*). We note that full details of the data analyses and the biological interpretations of the results are available elsewhere (Chambert et al. 2013) and so not discussed here. Our purpose in this paper is to further discuss this model-checking inferential approach to promote stronger biological inferences about individual heterogeneity in population vital rates. We particularly emphasize the choice of relevant data features and graphical displays used as informative model diagnostics. We finally discuss how we think this approach could benefit scientific progress in ecology.

## General Principle of Posterior Predictive Checking

Posterior predictive checks are a method of model checking based on comparing the distribution of random draws of new data generated under a specific model of interest to the observed data (Gelman et al. 2004). This approach simply relies on the intuitive idea that if a model is a good fit to the data, then the replicated data (*y*^*rep*^) predicted from that model should look similar to the observed data (*y*^*obs*^). To simplify the comparison, a summary discrepancy measure *T*(*y*), calculated from the data (*y*^*rep*^ and *y*^*obs*^) and relevant to the question of interest, is generally used. In practice, a posterior predictive check is implemented as follows: After a full probability model (likelihood and prior) has been specified and the posterior distribution of the parameters sampled, (e.g., using a MCMC algorithm; Gelman et al. 2004; Link and Barker 2010), we draw many replicate data sets *y*^*rep*^, calculate *T*(*y*^*rep*^) for each one, and then use a histogram to display the approximate distribution of *T*(*y*^*rep*^). This distribution is called a posterior *predictive* distribution and can also be used, assuming the validity of a model, to predict future or missing values (Link and Barker 2010). The observed quantity of interest *T*(*y*^*obs*^) is compared with this distribution to assess its plausibility under the model of interest and to therefore evaluate potential lack of fit of the model. Parameter uncertainty is explicitly accounted for because the data realizations are generated from parameter values randomly drawn from the posterior distribution. This is distinguished from investigations using model-generated data realizations where the parameters are set at single values (e.g., maximum-likelihood estimates) and thus do not incorporate parameter uncertainty.

The quantities *T*(*y*) used for comparison can be any feature derived from the data, or from a combination of data and parameters (*T*(*y,θ*)), and should be chosen according to the scientific context and question of interest (Gelman et al. 2004; Gelman and Shalizi 2013). The most useful test quantities are those that are not directly modeled, but are still of biological relevance for the question at hand (e.g., lifetime reproductive output). Graphical comparisons are a powerful way to highlight systematic discrepancies between the model predictions and observed data (Gelman 2003) and provide useful information on where the model is performing poorly and how the model can be improved. Posterior predictive *P*-values, defined as *Pr*[*T*(*y*^*rep*^) ≥ |*T*(*y*^*obs*^)|], can easily be calculated as a summary statistic of lack of fit for the chosen discrepancy measure (Rubin 1984; Meng 1994; Gelman and Shalizi 2013). Reservations about the use of posterior predictive *P*-values for model comparison have been expressed (Hjort et al. 2006), and we agree they should not be used in conjunction with an arbitrary threshold, like the classic Type I error *α* = 0.05, to simply reject or retain a given model. Posterior predictive *P*-values are, however, legitimate and informative probability statements (Gelman and Shalizi 2013) that are simply interpreted as the proportion of data replications under the specified model resulting in a value *T*(*y*^*rep*^) that exceeds *T*(*y*^*obs*^). In more intuitive words, Gelman (2013, p.3) explained that, “if a posterior predictive *P*-value is 0.4, that means that, if we believe the model, we think there is a 40% chance that tomorrow's value of *T*(*y*^*rep*^) will exceed today's *T*(*y*^*obs*^).” Here, we simply consider them as a numerical summary measure of the graphical comparison between *T*(*y*^*obs*^) and the distribution of *T*(*y*^*rep*^).

## Use of Posterior Predictive Checks as an Inferential Tool

Posterior predictive checks were initially developed to assess the goodness of fit of statistical models (Gelman et al. 1996), but they have also been used for exploratory data analyses and inferential purposes (Gelman 2003). The efficient use of posterior predictive checks relies on a few simple principles. First, the choice of informative test quantities *T*(*y*) depends on the specific context of a study and requires careful thinking beyond simple omnibus tests. Second, the use of graphical displays should be emphasized over posterior predictive *P*-values alone, as they provide more information about the lack of fit of a model. The *P*-values should simply be used as numerical summaries of the information captured in graphical displays. Finally, posterior predictive *P*-values, as well as graphical checks, should not be interpreted as universal values or tests independent of the context in which analyses are performed.

Here, we adopt the philosophy of Gelman and Shalizi (2013), admitting a priori that our models are wrong and using model checking as a tool to highlight their limitations and find ways to improve them. Posterior predictive checks can be efficiently used in an iterative process of model specification, checking, and refinement. In the context of individual heterogeneity in animal population vital rates, although we might readily admit a priori that individuals are not homogeneous, we are typically truly interested in quantifying the magnitude of underlying individual heterogeneity and assessing its biological importance. In their article on dynamic heterogeneity, Tuljapurkar et al. (2009) were not suggesting that wild animal populations can be entirely homogeneous but rather that underlying heterogeneity could sometimes be relatively weak, such that the majority of realized individual differences could be explained by chance (stochasticity from the binomial model). This question of whether the magnitude of individual heterogeneity is large enough to be practically meaningful can be directly addressed with posterior predictive checks based on the comparison of relevant quantities *T*(*y*) measuring variation in realized individual performance (see section Illustration with a Real Data Set for a concrete example for a concrete example). We can indeed use such checks to directly investigate how a model ignoring underlying individual heterogeneity might fail at predicting the levels of realized variation in selected individual performance measures. Similarly, we can directly assess whether a model that includes fixed individual random effects (e.g., Cam et al. 2002) provides a good approximation of the heterogeneity pattern prevalent in a population. In the spirit of model refinement, if some data features are still poorly predicted by this last model, this should lead us to build a third model, including, for example, some type of interactions between individual effects and environmental conditions (see example below). By simply comparing the first two models in a model-selection framework, it would be more difficult to assess the consequences of ignoring individual heterogeneity or gain understanding about how biologically meaningful the magnitude of heterogeneity is. Furthermore, posterior predictive checks provide a systematic way of verifying a model's adequacy at predicting important data features, which allows checking whether inferences from the posterior distribution are sensible and valid.

## Illustration with a Real Data Set

### Overview of the biological context and modeling

To illustrate the use of posterior predictive checks in a real case study, we use model results of a previous study (Chambert et al. 2013) that was motivated by two ecological questions: (i) the magnitude and (ii) the temporal stability of underlying individual heterogeneity in reproductive rates of female Weddell seals (*Leptonychotes weddellii* Lesson). Chambert et al. (2013) used posterior predictive checks to draw inferences and make biological conclusions, but they did not have the space to develop the key aspects and issues related to this approach, which is relatively new to ecologists. The goal of this study was to motivate the use of posterior predictive checks as an inferential tool in a broader set of study systems and ecological questions. Here, we therefore develop the underlying concepts of this method well beyond what is provided in the biology-focused paper and provide additional details on a number of key issues. Given that a full treatment of the modeling and biological interpretation of the results is provided in Chambert et al. (2013), here, we only provide a brief summary of these aspects and, instead, focus the discussion on the implementation and interpretation of posterior predictive checks.

Analyses were based on long-term mark–recapture data collected from 1982 to 2011 in a population of Weddell seals breeding in Erebus Bay, Antarctica. Reproductive rates were modeled as probabilities of transitioning into a breeder state using a multistate modeling approach (Williams et al. 2002), implemented in a Bayesian framework using program OpenBUGS (Lunn et al. 2009; Kéry and Schaub 2012). Individual heterogeneity was defined as the residual variance in individual reproductive rates after accounting for age, breeding state at *t−1*, and year, which are known sources of heterogeneity (Hadley et al. 2006, 2007; Rotella et al. 2009). To address the two questions of interest, three models were investigated: the first (*M1*) model assumed no individual heterogeneity in reproduction probabilities; the second model (*M2*) included individual random effects on reproduction probabilities assumed to be constant over time; and the third model (*M3*) extended this to allow individual effects to vary with environmental conditions (“normal” vs. “adverse”; Chambert et al. 2012). In models *M2* and *M3*, individual random effects were assumed to have a zero-centered normal distribution, and the magnitude of individual heterogeneity was quantified by the variance of this distribution. Two independent individual effects were included in model *M3*, one being expressed in normal years only and the other in adverse years only.

### Implementation of posterior predictive checks

After having approximated model-specific posterior distributions of parameters, we implemented posterior predictive checks, for each model, as follows: Using draws from the joint posterior distribution of all parameters, we simulated 10,000 replicate data sets (*y*^*rep*^, matrices of individual reproductive histories) with the same number of individuals, number of observations, and overall structure as that found in the original data set. Individual reproductive histories were simulated using year- and individual-specific reproductive rates calculated from the relevant set of parameters of each model. Random effect values (i.e., individual effects and year effects) were directly drawn from their posterior distribution, rather than being generated under assumed normal distributions, with mean and variance drawn from their posterior distributions. The posterior predictive checks were therefore conditional on the latent random effects directly. Given our questions of interest, we chose to compare quantities *T*(*y*) representing the levels of realized among-individual variation in reproductive performance, which were directly inspired by the work of Tuljapurkar et al. (2009) on dynamic heterogeneity. We used two measures of reproductive performance: (i) reproductive output (RepOutput), that is, the number of pups produced by a female within the study period; and (ii) the persistence in the breeder state (PersistRep), defined as the maximum number of consecutive of years an individual remained in the breeder state without skipping a year of reproduction (i.e., longest uninterrupted run of successive breeding years). As summary statistics of among-individual variation in these quantities of interest, we used the standard deviation (SD) and the maximum value (Max) across individuals. The four test quantities *T*(*y*) used were thus defined as: SD (RepOutput), Max (RepOutput), SD (PersistRep), and Max (PersistRep). Histograms were used to compare the posterior predictive distributions of each defined *T*(*y*^*rep*^) to the corresponding observed values *T*(*y*^*obs*^). One-sided (left-tail) posterior predictive *P*-values, calculated as the proportion of *T*(*y*^*rep*^) smaller than or equal to the observed value *T*(*y*^*obs*^), were used as a summary of the discrepancy between each model's posterior predictions and the observed data.

## Results

**Figure 1:**
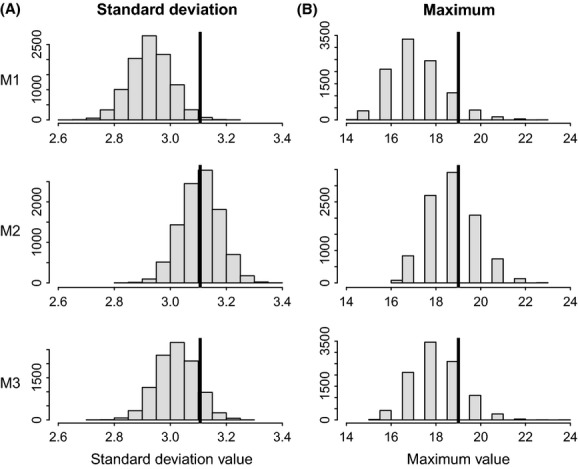
Posterior predictive distributions of (A) the standard deviation and (B) the maximum value, across individuals, for reproductive output. These distributions were obtained from 10,000 replicate data sets simulated from each competing model (*M1*,*M2*,*M3*), represented by each row in the graphs. The observed value from the real data is represented by a vertical black line.

**Figure 2:**
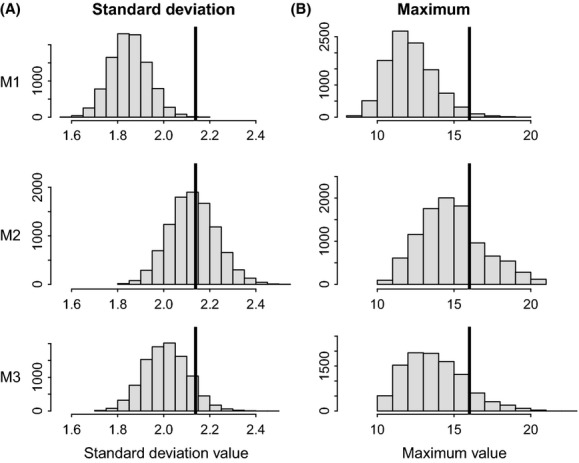
Posterior predictive distributions of (A) the standard deviation and (B) the maximum value, across individuals, for the time of persistence in the reproductive state. These distributions were obtained from 10,000 replicate data sets simulated from each competing model (*M1*,*M2*,*M3*), represented by each row in the graphs. The observed value from the real data is represented by a vertical black line.

Posterior distributions of the variances defining the distributions of individual effects in both heterogeneity models are clearly distinct from zero (see Chambert et al. 2013), which can be considered as enough evidence to conclude that individual heterogeneity exists in this population. However, this does not convey how this level of variability manifests in realized quantities of interest, such as those identified in the previous paragraph. The use of posterior predictive checks helps to further assess the practical relevance of the estimated magnitude of individual heterogeneity. Graphical displays of the posterior predictive distributions of the four test quantities (Figs 1 and 2) reveal that the model assuming homogeneity (*M1*) substantially underestimates the degree of individual variation in realized reproductive performance. All posterior predictive *P*-values are smaller than 0.12 for this model *M1* (Table 1). On the other hand, the two models that account for individual heterogeneity (*M2* and *M3*) predict levels of variation that are better aligned with the observed values (Figs 1 and 2). Posterior predictive *P*-values are between 0.35 and 0.49 for model *M2* and between 0.09 and 0.30 for model *M3*. These results indicate that the amount of realized individual variability observed in this population is not consistent with predictions from a model in which underlying individual heterogeneity in reproductive rates is ignored (assumed to be zero). Therefore, the observed variability cannot be solely explained by stochasticity in reproductive trajectories (dynamic heterogeneity). Another interesting biological implication of including individual heterogeneity in the model was also revealed by the detection of a reproductive cost on future reproduction that was not detected in the homogeneity model (Chambert et al. 2013). Furthermore, the more complicated model *M3* does not provide better predictions of quantities *T*(*y*) than model *M2*. This does not mean that the environment has no influence on the expression of individual heterogeneity, but simply that the complexity added in model *M3* does not noticeably improve model fit regarding the features of interest we identified, which summarize reproductive performance over a lifetime.

**Table 1:** Posterior predictive *P*-values for the standard deviation (SD) and maximum values (Max) of the two measures of individual reproductive performance: (i) reproductive output (RepOutput), and (ii) persistence in the reproductive state (PersistRep). See text for exact definitions of these variables. Posterior predictive *P*-values are provided for each of the three competing model: (*M1*) no individual heterogeneity, (*M2*) constant individual heterogeneity, and (*M3*) temporally variable individual heterogeneity. The values were obtained from 10,000 replicated data sets for each model.

Model	RepOutput		PersistRep	
	SD	Max	SD	Max
*M1*	0.007	0.115	0.001	0.032
*M2*	0.489	0.468	0.441	0.347
*M3*	0.108	0.296	0.089	0.184

**Figure 3:**
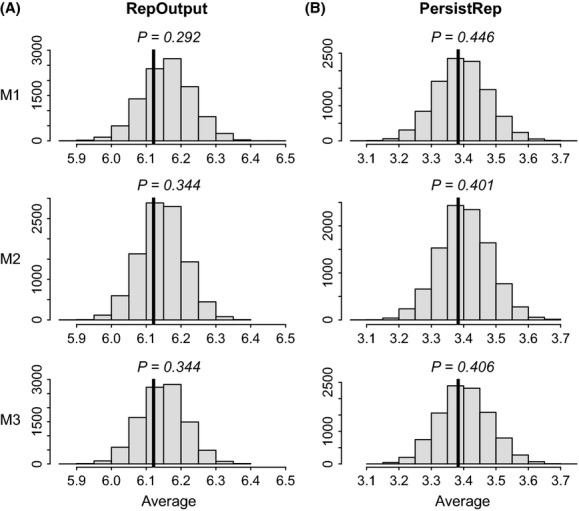
Posterior predictive distributions of the average value, over individuals, for the two measures of reproductive performances: (A) reproductive output (RepOutput); and (B) the time of persistence in the reproductive state (PersistRep). These distributions were obtained from 10,000 replicate data sets simulated from each competing model (*M1*,*M2*,*M3*), represented by each row in the graphs. The observed value from the real data is represented by a vertical black line. Corresponding one-sided posterior predictive *P*-values (*P*) are displayed on each histogram.

In a different study context, one could have decided to focus on different test quantities than the ones used here. There is obviously some subjectivity involved in these choices, but what is critical is to choose quantities that are informative. Here, because we were primarily tackling the dynamic heterogeneity question, we used the measures of variability in realized individual reproductive performances presented by Tuljapurkar et al. (2009). We also investigated the performance of the three models at predicting the average values of reproductive performance. All three models performed similarly well, with all posterior predictive *P*-values greater than 0.29 (Fig. 3). The use of this latter test quantity would thus not be sufficient to assess the importance of including individual random effects if one was interested in individual reproductive heterogeneity. On the other hand, if our goal was simply to estimate average reproductive performance values in the population, one could argue for using the homogeneity model, ignoring potential violations of independence. It is, however, important to remember that if individual heterogeneity is prevalent, but not accounted for, estimators of age- or state-specific parameters can be biased (Vaupel and Yashin 1985), as illustrated in our example with the reproductive cost question. Therefore, even if the focus is not on quantifying the magnitude of individual variation, it can still be relevant and useful to account for underlying individual heterogeneity.

## Discussion

We showed how posterior predictive checks could be used to strengthen inferences about the biological importance of individual heterogeneity in vital rates. In particular, this approach allowed us to directly address the dynamic heterogeneity issue by assessing whether a model assuming individual homogeneity in reproductive rates, but accounting for other relevant effects (age, year, and state), would predict levels of realized variation similar to those observed. The key was to use levels of variation (SD and Max) in measures of reproductive performance as test quantities *T*(*y*) of interest. Results showed that the homogeneity model largely underpredicted the magnitude of realized variation, indicating that additional underlying sources of individual variation were not captured by this model. This lack of fit had further implications of biological importance as an existing reproductive cost was not detected by this simple model (Chambert et al. 2013). When constant individual random effects were added, the new model predicted the levels of realized variation identical to the observed and the reproductive cost was evidenced. A more complex model considering the potential interaction of individual reproductive differences with environmental conditions did not provide better predictions of the features *T*(*y*) of interest. Other model improvements are certainly possible, but these results reveal that the model assuming constant heterogeneity provides sensible parameter estimates and can reasonably be used to draw inferences regarding individual variation in reproductive performances measured over a lifetime.

The relevance of posterior predictive checks for inferential purpose was shown here for individual heterogeneity issues, but this approach can easily be extended to other scientific questions focused on a particular pattern or process of interest (Gelman et al. 2004). For instance, studies on temporal variation in population vital rates often aim at investigating a priori hypotheses about environmental mechanisms underlying this variation. The support for models including environmental covariates of interest can be assessed by model selection, but this approach does not typically provide much information regarding what aspects of temporal variation are captured by the covariates and how much residual variation there is. Moreover, with large data sets, it often happens that a model including fixed or random year effects, rather than the covariate(s) of interest, will be better supported because of important residual variation, even if the covariate(s) of interest do(es) capture some of the variation. Such a result provides little useful information regarding the underlying mechanisms of primary interest. In such instances, posterior predictive checks could be used to strengthen inferences about the postulated underlying mechanisms by assessing the performance of various competing models at predicting relevant aspects of variation in the data. Furthermore, other checks could inform us on what aspects of variation the model is not capturing, which could help formulating new hypotheses about processes responsible for the residual variation. A new model or set of models could subsequently be built, and the new formulated hypotheses could then be further tested.

As illustrated by our example, posterior predictive checks can also be very useful to assess the implications of assumption violations, even when the assumption concerns a nuisance parameter of no direct biological interest, such as detection probability. Heterogeneity in detection probabilities has long been a concern in mark–recapture studies as it can induce biases in demographic parameter estimators, such as population size and survival (Buckland 1982; Pollock 1982). To deal with this issue, goodness-of-fit tests aiming at assessing the degree of violation of the assumption are usually used, but these hypothesis tests are very sensitive to sample size and do not provide any information on the real implications of assumption violation. Posterior predictive checks could be used, in this case, to directly assess the implications of the homogeneity assumption and suggest ways of modifying the model if the assumption violation seems problematic. Furthermore, because posterior predictive checks are not restricted to predefined model structures, they can be used to assess the fit of complex models (e.g., including age effects), which cannot always be carried out with classic goodness-of-fit tests developed for mark–recapture models (Williams et al. 2002).

The inferential approach developed in this paper is not meant to replace model selection, as these two approaches have a different focus and therefore different strengths and limitations. Indeed, while model selection is primarily aimed at discriminating among a priori competing models (Burnham and Anderson 2002), posterior predictive checks focus on highlighting the shortfalls of a specific model. Depending on a study's goal, one approach might be more appropriate than the other, but often both can be complementary and used in conjunction. For instance, in the presence of a large number of a priori competing models, it would certainly be more efficient to first use a model-selection approach to select a limited number of models. But then, posterior predictive checks could be implemented on these few best models to explore the biological meaning of each model's features and suggest ways to further improve them (Gelman 2003). We think that the use of posterior predictive checks, potentially in tandem with other methods, could help strengthen inferences and also enhance the process of model refinement. However, as with any other statistical approach, posterior predictive checks will not compensate for poorly designed studies. Strong inference can only be achieved by the careful implementation of appropriate study designs and the formulation of relevant a priori hypotheses. Nevertheless, we think that this approach, which can be implemented at any step of scientific investigation, from exploratory data analyses to inferences, including goodness-of-fit assessment and hypothesis generation (Rubin 1984; Meng 1994; Gelman 2003), will be a useful addition to the set of analytical methods used in ecology.

## Data Accessibility

http://www.montana.edu/rgarrott/antarctica/project_description.htm
